# Two complementing *in vivo* selection systems based on CCA-trimming exonucleases as a tool to monitor, select and evaluate enzymatic features of tRNA nucleotidyltransferases

**DOI:** 10.1080/15476286.2025.2453963

**Published:** 2025-01-20

**Authors:** Karolin Wellner, Josefine Gnauck, Dorian Bernier, Stephan H. Bernhart, Heike Betat, Mario Mörl

**Affiliations:** a Institute for Biochemistry, Leipzig University, Leipzig, Germany; b Bioinformatics Group, Department of Computer Science and Interdisciplinary Center for Bioinformatics, Leipzig University, Leipzig, Germany

**Keywords:** CCA-adding enzyme, tRNA nucleotidyltransferase, CCA-trimming exonuclease, RNase LCCR4, RNase T, *in vivo* screening of CCA-adding activities

## Abstract

tRNA nucleotidyltransferase represents a ubiquitous and essential activity that adds the indispensable CCA triplet to the 3’-end of tRNAs. To fulfill this function, the enzyme contains a set of highly conserved motifs whose coordinated interplay is crucial for the sequence-specific CCA polymerization. In the human enzyme, alterations within these regions have been shown to lead to the manifestation of disease. Recently, we developed an *in vivo* screening system that allows for the selection and analysis of tRNA nucleotidyltransferase variants by challenging terminal AMP incorporation into tRNA during induced RNase T-catalyzed CCA-decay. Here, we extend this method for screening of full CCA-end repair by utilizing the CCA-trimming activity of exonuclease LCCR4. To demonstrate the combined potential of these two *in vivo* selection systems, we applied a semi-rational library design to investigate the mode of operation of catalytically important motifs in the human CCA-adding enzyme. This approach revealed unexpected requirements for amino acid composition in two motifs and gives new insights into the mechanism of CCA addition. The data show the potential of these RNase-based screening systems, as they allow the detection of enzyme variations that would not have been identified by a conventional rational approach. Furthermore, the combination of both RNase T and LCCR4 systems can be used to investigate and dissect the effects of pathogenic mutations on C- and A-addition.

## GRAPHICAL ABSTRACT

**Figure:**
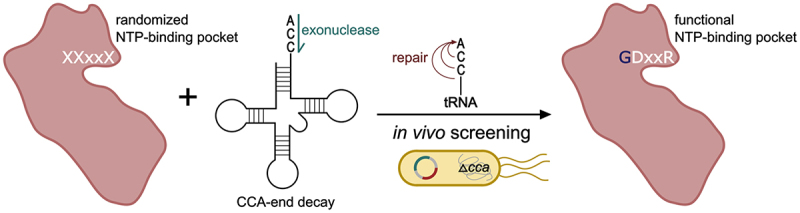

## Introduction

To carry out their role as adapter molecules in translation, tRNA primary transcripts undergo a series of processing steps in their maturation pathway, including the synthesis of the highly conserved CCA sequence at the 3’-end, representing the site for amino acid attachment [1]. The enzyme responsible for this processing step is tRNA nucleotidyltransferase (CCA-adding enzyme). In organisms where the CCA sequence is not encoded in the tRNA genes, this enzymatic activity is indispensable for cell survival [2]. According to their sequence and structure, these enzymes are grouped into class I (archaeal) and class II (bacterial and eukaryotic) enzymes [3–5]. In class II enzymes, a set of highly conserved motifs A to E make up the catalytic core in the N-terminal region (Figure S1) [6]. Motif D (EDxxR; x, any amino acid) is of special interest, as it functions as a template that – depending on the orientation of the side chains of arginine and aspartate – forms specific hydrogen bonds to the incoming nucleotide [6,7]. As the same templating residues have to interact with both CTP and ATP, the switch from C to A recognition/addition requires a certain flexibility of the catalytic core to facilitate the required adjustment of the nucleotide-binding pocket during polymerization [8–10].

In addition to the *de novo* CCA synthesis, the CCA-adding enzyme restores and maintains damaged 3’-ends throughout the life time of a tRNA [11–13]. Furthermore, it scrutinizes tRNA molecules in a quality check and labels damaged tRNAs for degradation by adding a double CCA tag [14–17]. These functions in restoration and quality control illustrate as to why CCA-adding enzymes are preserved in evolution even in organisms where the CCA-end is encoded in the tRNA genes [12,18]. Furthermore, an interplay of CCA-adding enzyme and CCA-trimming activities like RNase T, angiogenin, tRNase Z (the *E. coli* homologue is RNase BN) or the recently identified LCCR4 from *Trypanosoma brucei* function as a rapid and reversible way to shut down translation and, consequently, cell growth under certain environmental conditions [16,19–23].

Throughout the past decade, an increasing number of cases has emerged that associate alterations in human CCA-adding enzyme (*Hsa*CCA) with a hypomorphic phenotype and disease manifestation (reviewed in [24,25]. While therapeutics are on the way to assist in rendering the genetic disorder more manageable [26], continual efforts are needed to uncover the impact of such alterations on the catalytic properties of this enzyme. Here, *in vivo* screening systems are an advantageous approach to maximize the analytical toolbox when evaluating such enzyme variants.

In *E. coli*, the investigation of tRNA nucleotidyltransferases can be achieved in the strain CA244 Δ*cca* carrying an amber mutation within *lacZ* which can be read through in the presence of a functional suppressor tRNA^Tyr^ [27–29]. The system relies on the exogenous expression of both the tRNA nucleotidyltransferase of interest as well as su3 tRNA^Tyr^ lacking its 3’CCA tail. Alternatively, the antagonistic behaviour of tRNA-specific 3’-exonucleases can be utilized to provide a substrate pool for CCA-restoring activities. We have recently presented such an *in vivo* method that enables screening and selecting for tRNA nucleotidyltransferase activity by challenging tRNA 3’-end repair during enforced RNase T-catalyzed CCA degradation in *E. coli* JM109(DE3) Δ*cca* [30]. This approach coupled CCA-adding efficiency directly to *E. coli* growth fitness and was used to evaluate the catalytic activity of a reconstructed ancestral enzyme candidate under natural conditions [31]. While this method represents a very useful analytical approach, a limitation of the system is that it is restricted to terminal AMP incorporation and does not address the full CCA-addition.

Here, we present an extension of the method beyond that limitation. Exploiting the CCA-trimming activity of LCCR4 from *T. brucei*, we use an *E. coli* growth phenotype read-out to address CCA-specific tRNA repair activity. By means of a semirational library design, the functionality of two catalytically important elements in *Hsa*CCA was investigated. This approach revealed a strong demand for an arginine residue at position 105 within the flexible loop region and a variation of functionally compatible amino acids compensating for the conserved glutamate at position 164 in the amino acid template of motif D (Figure S1, numbering according to [10,32–34]). The variants show efficient CCA and A repair activity both *in vivo* as well as *in vitro*. Furthermore, the enhanced *in vivo* screening system allowed for the characterization of the unexpected substitution E164G, demonstrating the potential to explore so far unknown catalytic properties in CCA-adding enzymes. Finally, the combination of both screening approaches can be used to characterize the functional defects of human CCA-adding enzyme variants carrying pathogenic amino acid replacements.

## Materials and methods

### Bacterial strains, media and growth conditions

Bacterial strains used in this study were *E. coli* BL21(DE3) (Novagen) and JM109(DE3) (Promega). Disruption of the *cca* gene (*cca:cam*, ∆*cca*) was achieved as described [30]. *E. coli* TOP10 was used for cloning and mutagenesis experiments. Routinely, *E. coli* cells were grown at 37°C in LB or TB supplemented with antibiotics where needed at the following concentrations: kanamycin (kan) 30 µg ml^−1^; chloramphenicol (cam) 34 µg ml^−1^; ampicillin (amp) 100 µg ml^−1^. To determine relative RNase T or LCCR4 tolerances due to CCA-adding activity, cells were spread on agar gradient plates. The plates were prepared with a layer of sloped LBamp agar containing 25 to 200 µM IPTG, depending on the screening system. After solidifying as a wedge, a second LBamp agar layer without IPTG was poured on the first slope. Vertical diffusion of IPTG into the upper wedge established a linear gradient in a range proportional to the thickness of the IPTG-containing wedge. For 24 h of bacterial growth, the IPTG gradient is constant and linear across the plate (ranging from 0 to the maximum concentration in the lower wedge), so that the induced RNase expression is correspondingly increasing. The usefulness of such gradient plates was demonstrated for minimal inhibitory concentrations of antibiotics as well as enzyme activities [30,31,35,36].

### Cloning of vector constructs, DNA library generation and *in vivo* selection

Routinely, the respective RNase ORFs were cloned between the *Eco*RV and *Avr*II restriction sites (in MCS2) of the previously described pETDuet1 construct [30]. ORFs of nucleotidyltransferase variants were introduced into the autonomous expression platform of MCS1 via site-directed mutagenesis.

The pETDuet-derived vector libraries were generated utilizing a set of PAGE-grade purified oligonucleotides containing degenerate codons (biomers.net). Site saturation mutagenesis with NNK codons was used when generating the 105NNK position in the RNase T system, while the 22c trick method [37] was applied when preparing the library at the same position in the LCCR4 system. In the latter case, *Dpn*I digest preceded gel extraction and overlap extension PCR to generate an insert out of the amplified DNA fragments (Figure S2). NEBuilder HiFi DNA assembly (New England Biolabs) was used for the insertion into the respective vector background after linearization of the vector DNA via Q5 DNA polymerase (New England Biolabs) and *Dpn*I digest. The same procedure was carried out when introducing the 22c trick degeneracy at the position 105 in combination with position 164. When partially randomizing all three positions 164, 165 and 168 in motif D, RVK codons were used in place of the 22c trick approach. Sequences of the used oligonucleotides are listed in Table S1. To investigate possible biases in the generated libraries, the relevant ORF regions of the assembled vector libraries were amplified and subjected to Illumina paired end Amplicon sequencing (GENEWIZ). In the about 96,000 pairs sequenced, both randomized codon positions could be identified in 68,496 (read 1, representing 71%) and 59,920 (read 2, 62%) of the reads. Reads were searched and counted in Perl, using regex patterns of the flanking sequences, analysis was done by R [38]. At codon position 105, Arg (R; CGT) was present in 2% of read 1 and read 2, with the abundance of all 22 codons inserted probably normally distributed (Shapiro test *p* > 0.8, see Figure S3A). As for position 164, we analysed the abundance of the codons for E (GAG), D (GAT) and G (GGT) in all sequenced reads as well as in the subset of reads that had an arginine codon at position 105, as we show this amino acid to be a prerequisite for a functional CCA-adding enzyme (see Results section). For all reads, the abundance of the three codons was between 5% and 6%. For the much lower number of reads containing R105, the percentages varied slightly more, showing an abundance of 4% to 7% (see Figure S3B). However, while these codons are among the more frequent ones, even combined they stay below 20% abundance and probably belong to a normal distribution (Shapiro test results ranging from *p* = 0.3 to *p* = 0.7).

The generated DNA product mix as well as plasmids containing individual ORFs for nucleotidyltransferase variants were directly introduced into *E. coli* JM109(DE3) ∆*cca*. Resulting transformants were plated onto LB agar containing ampicillin and indicated IPTG concentrations, single colonies were analysed after overnight incubation at 37°C and subsequently sequenced.

Pathogenic point mutations were introduced into the expression plasmids by Q5 mutagenesis and verified by sequencing of individual clones.

### Overexpression and purification of recombinant enzymes

Recombinant *Hsa*CCA variants with N-terminal His-Tag were overexpressed from pET28a(+) in *E. coli* BL21(DE3) *cca:cam* cells lacking the endogenous CCA-adding enzyme. Cells were grown in TBkan/cam at 30°C until an OD_600_ of 1.5. Overproduction was induced by the addition of 1:2 cold induction medium to a final concentration of 1 mM IPTG. Cells were harvested after further incubation for 16 h at 16°C.

Cell pellets were resuspended in ice-cold lysis buffer containing 25 mM HEPES/KOH (pH 7.5), 500 mM NaCl, 1 mm DTT and 0.2 mM PMSF. Cells were disrupted, and recombinant enzymes were purified via HisTrap FF 1 ml as previously described [39] except for using 25 mM HEPES/KOH (pH 7.5) and 500 mM NaCl as binding buffer and HiLoad 1660 Superdex 75 pg (Cytiva) with the described binding buffer containing 200 mM NaCl. Protein-containing fractions were pooled, concentrated with Vivaspin 6 columns (Sartorius) and stored either in 40% glycerol (v/v) at 20°C for nucleotide incorporation assays or in 10% glycerol (v/v) at −80°C for DRaCALA assays.

### Preparation of in vitro tRNA substrates and analysis of reaction product 3’ ends

Radioactively labelled yeast tRNA^Phe^(GAA) substrate was synthesized via *in vitro* transcription in the presence of α ^32^P ATP (Hartmann Analytic) and purified as described [40,41].

To analyse the 3’ sequence after *in vitro* nucleotide incorporation, a 5’phosphorylated and 3’blocked tagging oligonucleotide (5’pUGGATCGCGTAGCTCATACGAGT3’3’T) was ligated to the tRNA using T4 RNA ligase [42]. cDNA was synthesized using SuperScript IV reverse transcriptase (Invitrogen) and a DNA reverse primer (5’AACTCGTATGAGCTACGCGATC3’) complementary to the tagging oligonucleotide. The cDNA was purified by phenol/chloroform extraction and ethanol precipitation [43]. In a standard PCR reaction, the purified cDNA was amplified using the above-mentioned reverse primer and a forward primer specific for yeast tRNA^Phe^ (5’GCGGATTTAGCTCAGTTGG3’). PCR products were cloned using the CloneJET PCR cloning kit (Thermo Fisher Scientific) and single colonies were sequenced.

### In vitro nucleotide incorporation assay

Nucleotide incorporation assays with recombinant enzymes (75 pg µl^−1^) were performed in a 20 µl reaction volume as described [30] at indicated incubation times in the presence of 1 mM of each NTP or individual GTP, UTP, CTP or ATP, respectively. For densitometric quantification of timeresolved product formation, master mixes were preheated to 37°C for 5 min prior to the reaction start.

### Analysis of nucleotide binding behaviour of HsaCCA and HsaCCA E164G

To investigate the CTP and ATP binding behaviour of the human CCA-adding enzyme and its variant E164G, the DRaCALA approach (differential radial capillary action of ligand assay) was used according to Roelofs et al. [44]. In a total volume of 10 µl, enzyme concentrations from 0 µM to 80 µM were incubated at 4°C with 1 nM of either α ^32^P ATP or α ^32^P CTP (Hartmann Analytic) in 30 mM HEPES/KOH (pH 7.6), 30 mM KCl, 6 mM MgCl_2_. The binding reaction was stopped after 10 min by spotting 2 µl of each sample onto an Amersham^TM^ Protran^TM^ nitrocellulose membrane 0.45 µm (Cytiva). For each enzyme, 3 technical replicates of 3 biological replicates were performed. Due to the limited solubility of the enzymes, binding constants could not be determined.

## Results

### CCA-trimming RNases can serve as selective factors for proper CCA-addition

While the *E. coli* exonuclease RNase T only removes the terminal A residue of the tRNA CCA-end [16,45], RNase LCCR4 (*T. brucei*) exhibits efficient trimming of the complete CCA sequence [23]. Furthermore, the CCA-removing activity of the *E. coli* enzyme RNase BN is controversially discussed [22,46]. Hence, both activities were tested whether they can be used for expanding our *in vivo* complementation system for A addition [30] into an approach that monitors the complete CCA-adding activity of a tRNA nucleotidyltransferase of interest. To exclude any endogenous CCA-adding activity that could skew the results, all *in vivo* experiments as well as recombinant enzyme productions were carried out in *E. coli* strains where the gene for the genomically encoded CCA-adding enzyme was knocked out [30].

**Figure 1.**
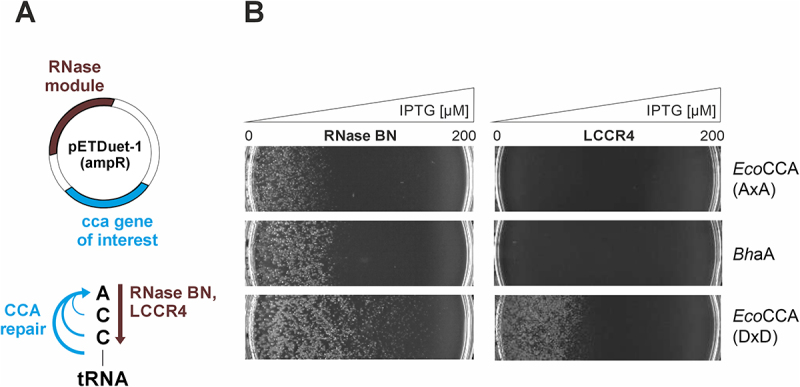
Coexpression of CCA-trimming exonucleases can be used to monitor the CCA-adding activity of recombinantly expressed tRNA nucleotidyltransferases *in vivo*. (A) Principle of the *in vivo* system is based on a pETDuet-derived vector enabling dual expression of an RNase (brown) and a tRNA nucleotidyltransferase of interest (cyan), subjecting the tRNA pool in *E. coli* JM109(DE3) Δ*cca* to CCA-end hydrolysis and restoration. (B) *E. coli* growth was examined on LBamp plates in an IPTG gradient from 0 to 200 µM, resulting in a gradual increase of tRNA-CCA end hydrolysis mediated by recombinant co-expression of either *E. coli* RNase BN (left) or *T. brucei* LCCR4 (right). Only enzymes with full CCA-adding activity give rise to selective bacterial growth. Removal of CCA-ends by RNase BN results in efficient growth of cells coexpressing the wt CCA-adding enzyme. Co-expression of the inactive enzyme variant *Eco*CCA (AxA) or the A-adding enzyme *Bha*A also lead to cell growth, although at a clearly restricted efficiency. This is an indication that under RNase BN expression, a considerable number of tRNAs still carry a complete or partial CCA-end, interfering with a selective growth depending on CCA-adding activity. *Eco*CCA, *E. coli* CCA-adding enzyme; DxD, wildtype enzyme with catalytically active carboxylates; AxA, inactive enzyme variant with catalytic carboxylates replaced by alanine residues; *Bha*A, *B. halodurans* A-adding enzyme.

When complementing with a set of tRNA nucleotidyltransferases in *E. coli* JM109(DE3) Δ*cca*, a distinct growth phenotype was observed, correlating with a gradual increase in overexpression of both exonucleases in IPTG gradient plates, respectively. *E. coli* wildtype CCA-adding enzyme *Eco*CCA DxD (DxD represents the two catalytically essential aspartate residues at positions 21 and 23 in the active site of the enzyme [30]) compensated for RNase BN stress up to a level of approximately 140 µM IPTG, while the inhibitory concentration for LCCR4-mediated stress could be attributed to about 80 µM (Figure 1). In the RNase BN system, the complementation with the A-adding enzyme of *Bacillus halodurans* (*Bha*A) or an inactive variant of the *E. coli* enzyme (*Eco*CCA AxA, where the catalytic aspartate residues were replaced by alanine [30]) still allowed for cell growth at moderate IPTG concentrations, indicating that this RNase did not efficiently remove full CCA-ends from the tRNA pool. In contrast, LCCR4-induced tRNA 3’end damage required the full CCA-adding activity of the complementing enzyme, as only *Eco*CCA DxD (wt) rescued bacterial growth on the gradient plate. Therefore, we decided to proceed with LCCR4 at a minimal IPTG concentration of 10 µM to challenge CCA repair.

### Site saturation mutagenesis uncovers the stringent identity at position 105

**Figure 2.**
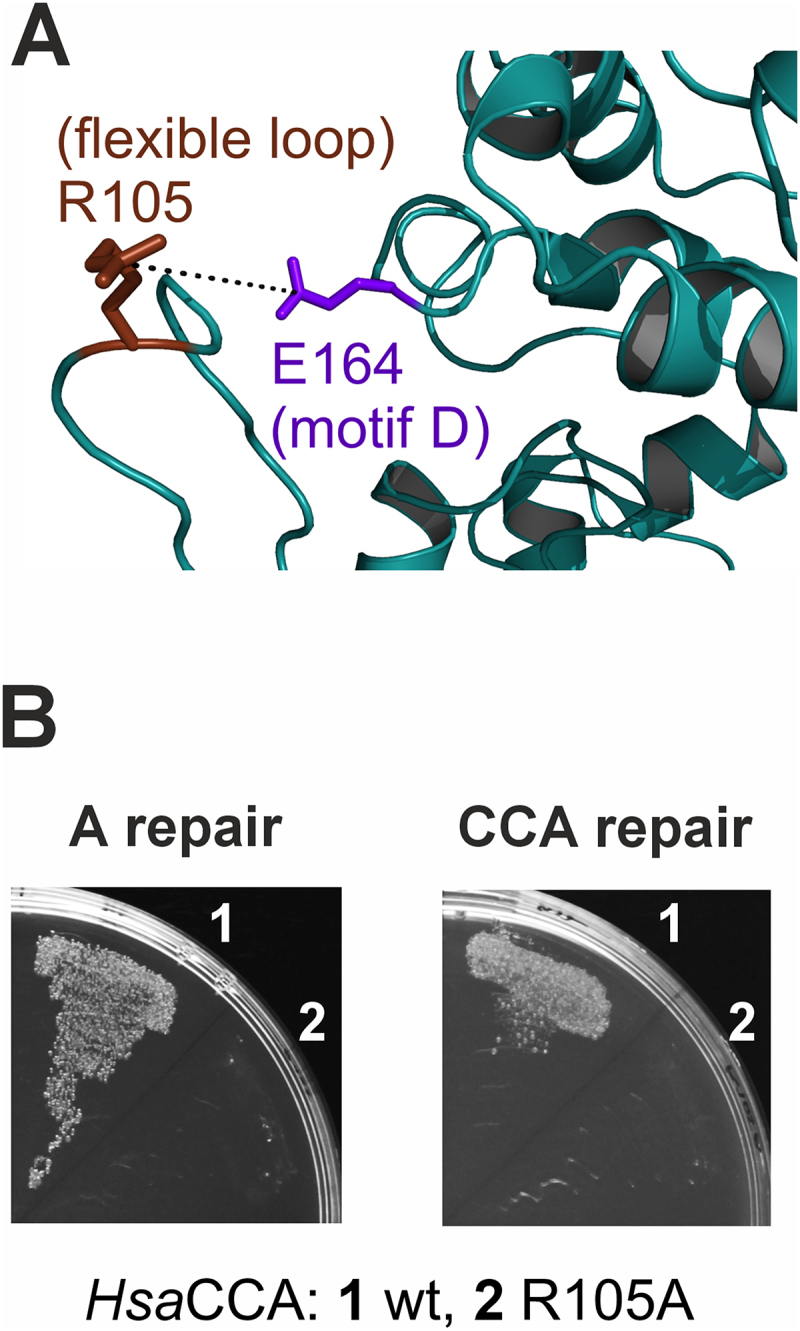
*Hsa*CCA R105A is severely impaired in full CCA addition. (A) As previously hypothesized [10,47], a salt bridge could be formed between R105 in the flexible loop region and E164 in motif D to switch the enzyme’s specificity from CTP to ATP incorporation. The presented model of the protein region including the flexible loop is based on the crystal structure of *Hsa*CCA (pdb entry 1OU5) according to Hoffmeier et al. [10]. (B) Complementation of *Hsa*CCA R105A (2) does not promote growth when selecting for A- or cca-repair activity, while the wildtype (wt) enzyme (1) rescues the growth defect.

To test the robustness and reliability of the LCCR4-based selection system for CCA-addition, we focused on a highly conserved position in the essential flexible loop element of the well-characterized human CCA-adding enzyme *Hsa*CCA. Depending on the sequence context of the individual enzymes, this loop region exhibits an extraordinary evolution into different sequence families that are conserved within the individual bacterial and eukaryotic branches of the phylogenetic tree [10]. An arginine within the loop at position 105 is essential for switching the enzymes’ specificity from C- towards A-addition, and it is hypothesized that it forms a salt bridge to glutamate 164 in the amino acid template of motif D, acting as a lever to adjust the nucleotide-binding pocket for accommodation of ATP (Figure 2A, S1) [10,47]. Consequently, the replacement of R105 by alanine (R105A) almost completely abolished A-addition in the corresponding *in vitro* experiments (1% compared to the wt enzyme), while the CC incorporation remained unaffected [10]. The *in vivo* selection system corroborates these *in vitro* results, as cells expressing a human CCA-adding enzyme with the described R105A replacement are not viable when RNase T (A repair) or LCCR4 (CCA repair) is co-expressed. The wt *Hsa*CCA, however, restores full CCA-ends, resulting in cell growth under both A as well as CCA repair (Figure 2B).

**Figure 3.**
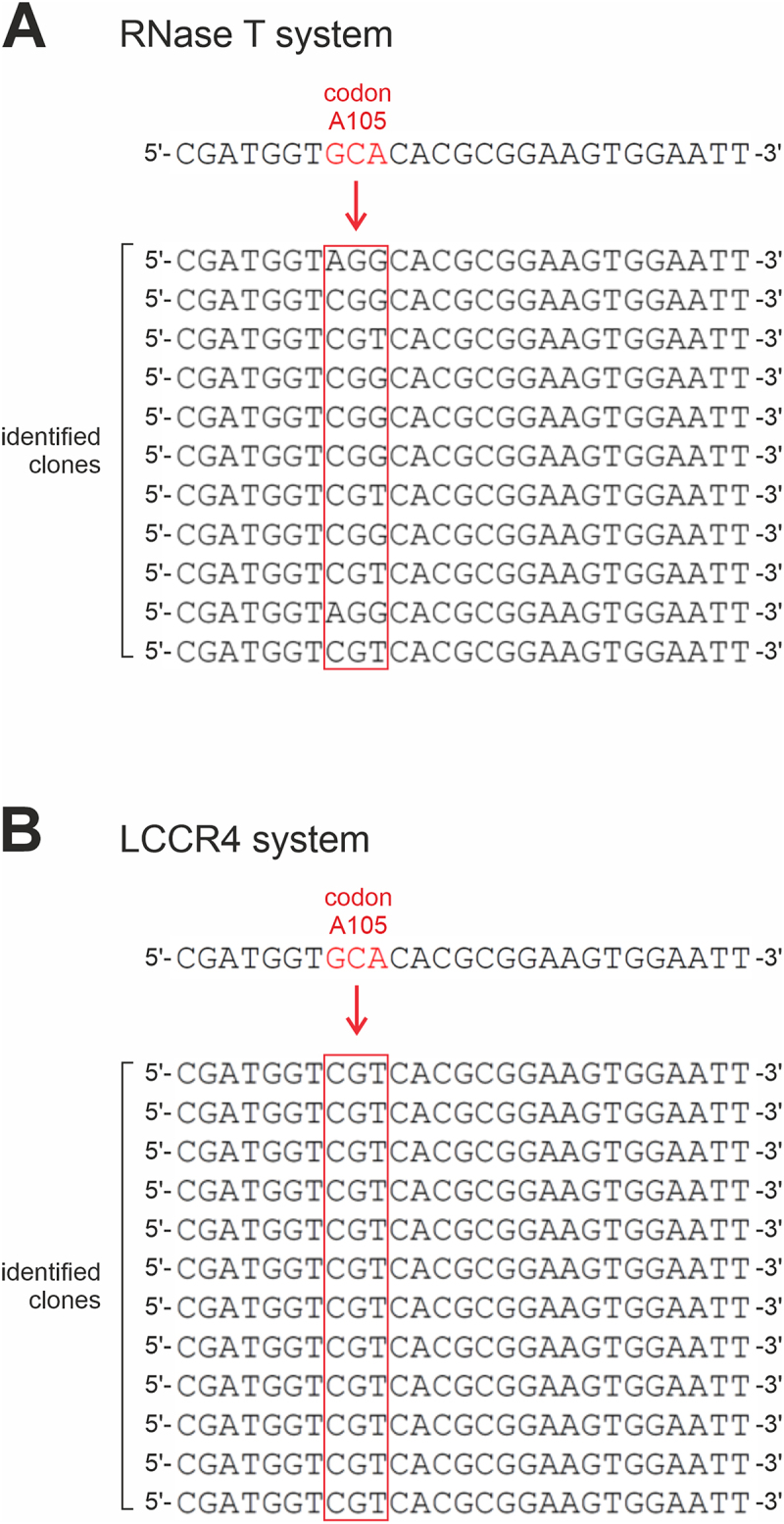
*In vivo* selection of position 105 in *Hsa*CCA. Codon 105 was randomized in the open reading frame of *Hsa*CCA. The resulting pool was subjected to selection in both RNase T-based (A) as well as LCCR4-based selection system (B). Under both conditions selecting for A- or CCA-addition, only arginine codons could be retrieved, indicating that this residue is invariant and essential for complete CCA-addition. As the NNK (K = G, T) randomization in the RNase T system includes the arginine codons CGT, CGG and AGG, all these codons could be selected. In the LCCR4 system, the 22c-trick mutagenesis was applied. This includes only one (CGT) arginine codon. Hence, only this codon appeared in the selection. To avoid any artificial arginine retrieval, the starting sequence for randomization contained an alanine codon GCA at this position (red). Hence, a selection bias due to residual wt sequences can be excluded. For both selections, a representative number of individual clones is shown.

Based on the A-adding deficiency of *Hsa*CCA R105A, we subjected position A105 to site saturation mutagenesis and introduced the generated DNA pool into JM109(DE3) Δ*cca*. After *in vivo* selection under RNase T (monitoring A-addition) or LCCR4 expression (monitoring CCA-addition), the resulting colonies were screened for appropriate nucleotide incorporation activity, and the corresponding *Hsa*CCA ORFs were sequenced. For both A as well as CCA repair selection, identical results were obtained. Out of all possible codons for the 20 amino acids, we exclusively retrieved arginine codons at this position, reflecting the wildtype situation (Figure 3). Interestingly, when providing NNK codons for degeneracy, all three possible codons for arginine could be retrieved, a further indication of a very low bias in the NNK distribution in the library. The strong selection for R105 suggests a highly essential nature of this position to yield an efficient A- or CCA-adding activity, respectively. Furthermore, a selection artefact due to mutations within the expressed RNase ORF could be excluded, as the integrity of the corresponding sequence in the propagating pETDuet construct was verified by sequencing.

### Semirational library design reveals an unexpected compensation for amino acid templating

**Figure 4.**
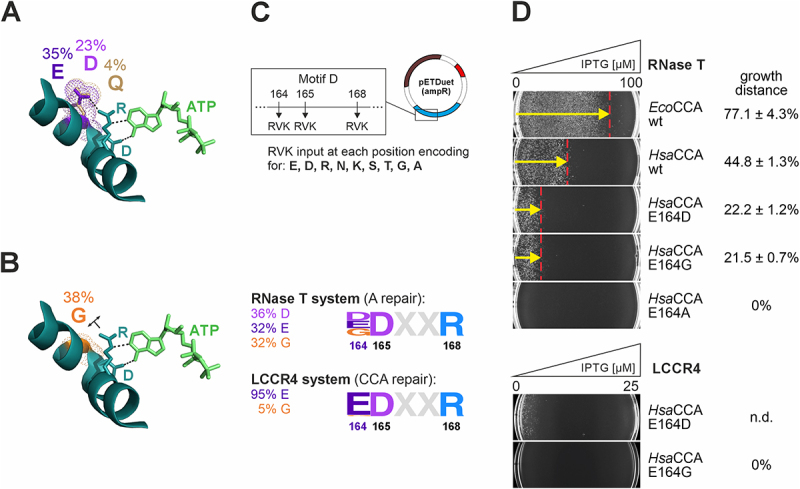
Semirational library design uncovers *Hsa*CCA enzyme variants with altered active site residues capable of efficiently repairing tRNA 3′ends. (A) Amino acid template in motif D of class II CCA-adding enzymes and its specific interaction with ATP (green). The model is based on the co-crystal structure of *Gst*CCA, the CCA-adding enzyme from *Geobacillus stearothermophilus* (1MIW) [6]. ATP and relevant amino acid residues are presented as stick models, hydrogen bonds are shown as black dashed lines; the structural surrounding was omitted for reasons of clarity. In the library selection with randomized positions 105 and 164, asp (D), glu (E) and gln (Q) appeared at position 164 (Figure S4). These residues (with their respective frequency at which they were retrieved) were placed by the PyMOL mutagenesis wizard and were further highlighted in dot representation. (B) In the same selection, glycine (G) appeared at position 164. In this situation, the templating side chain of R168 has a considerably increased positional freedom (indicated by the double arrow) that reduces the specificity of the NTP binding pocket. (C) After introducing a DNA library with tailored codon diversity at the three conserved template positions 164, 165 and 168 in *Hsa*CCA motif D, the retrieved sequences from the RNase T selection system – and to a much lower extent from the LCCR4 system – show alternative amino acid side chains only at position 164 (Figure S5). Letter heights in the sequence logos correlate with frequency at which they were retrieved after selection. As the randomized library contained between 5 and 6% E, D and G codons, respectively, this result represents a clear codon enrichment based on enzyme function. Sequence logos were created using the WebLogo generator [49]. (D) Growth of *E. coli* JM109(DE3) Δ*cca* complemented with the retrieved *Hsa*CCA variants was assessed on LBamp in an IPTG gradient from 0 to 100 µM, resulting in a gradual increase of RNase T-mediated tRNA end hydrolysis. A representative series of gradient plates out of 3 independent experiments is shown. Cells expressing the control enzyme *Eco*CCA wt tolerate very high RNase T stress, resulting in colonies over 77.1 ± 4.3% of the IPTG gradient distance. *Hsa*CCA wt is less active, but still results in considerable RNase T tolerance, allowing cell growth up to 44.8 ± 1.3% of the gradient. Variants E164D and E164G are viable at low RNase T expression levels (E164D: growth distance of 22.2 ± 1.2%; E164G: 21.5 ± 0.7%), while variant E164A is not viable at all, even at the lowest RNase T levels.

As there are indications that R105 forms a salt bridge to E164 in the amino acid template to adjust the nucleotide-binding pocket for the different NTP specificity during CCA addition [10,47], we hypothesized that possible variations at position 105 might be constrained when the interaction partner E164 remains unchanged. Hence, we performed a selection experiment based on a combinatorial DNA library with a simultaneous full amino acid diversity at both positions 105 and 164. Conceivably, this approach could yield so far uncharacterized enzyme variants harbouring an alternative salt bridge reconstitution [48]. Yet, under the stringent RNase-induced selective pressure, the results did not show any alternative amino acid residues at position 105 (Figure S4). By contrast, position 164 was more tolerant regarding amino acid identity. When selecting for A-addition/repair, wildtype glutamate was retrieved in 35% of the sequenced clones, but also glycine (38%), aspartate (23%), and to a lesser extent glutamine (4%) were found (Figure 4A, B, Figure S4). While the compatibility of aspartate and glutamine at this position is likely based on charge and geometric dimension related to glutamate, respectively (Figure 4A), the emergence of the smallest amino acid glycine (Figure 4B) was rather unexpected. To reproduce and confirm these selection results, a further library was generated, where all three positions of the amino acid template EDxxR were randomized (Figure 4C). The starting ORF contained alanine residues at all three positions (AAxxA) and did not give rise to viable colonies in the selection systems that would represent false positive background signals. To ensure complexity and sufficient coverage of the generated DNA pool, the degenerate codon RVK was used to provide tailored diversity including a set of hydrophilic/charged amino acids (RVK leads to single codons for E, D, R, N, K and S, and two codons for G, T and A) at each respective position (the codon for position R105 remained constant). The subsequent *in vivo* selection for A-addition/repair (RNase T) reproduced the findings and uncovered both DDxxR (36% of the clones) and GDxxR (32%) in addition to wildtype EDxxR (32%). In the *in vivo* system monitoring CCA-addition/repair (LCCR4), predominantly EDxxR (95%), but also GDxxR (5%) could be retrieved (Figure 4C, Figure S5). Strikingly, amino acid variations replacing D165 and R168 were not obtained, suggesting that recognition of the incoming nucleotide by these positions is achieved through an uncompromising geometry of specific hydrogen bond formation.

To monitor the respective sensitivity to RNase-induced tRNA end turnover *in vivo*, we reintroduced the isolated sequences into JM109(DE3) Δ*cca* and incubated the transformants on IPTG gradient plates in both RNase T as well as LCCR4 system. In the RNase T system, transformants complemented with wt *Eco*CCA in the pETDuet construct were used as a positive control, since these cells are thriving up to an IPTG concentration of ~80 µM after 24 h incubation at 37°C (77.1 ± 4.3% of the total gradient distance; a representative example is shown in Figure 4D) [30]. The retrieved enzyme variants *Hsa*CCA E164D and E164G were both able to promote growth at low levels of RNase T expression (22.2 ± 1.2% and 21.5 ± 0.7% of the gradient, respectively. This corresponds to ~20 µM IPTG (examples are shown in Figure 4D) but showed a significant decrease in the minimal inhibitory IPTG concentration compared to *Hsa*CCA wildtype (44.8 ± 1.3% gradient distance; ~40 µM IPTG; *p* = 0.0001). In contrast, *Hsa*CCA carrying an alanine replacement (E164A) did not rescue a growth phenotype, in agreement with the fact that no such variants were retrieved in the *in vivo* selection procedures. In the LCCR4 system, however, only *Hsa*CCA E164D showed a minimal but not quantifiable growth at a reduced IPTG gradient concentration of maximum 25 µM (Figure 4D), while *Hsa*CCA E164G was not viable at all, in contrast to the selection screen, where 5% of the clones carried this replacement. However, the screenings were done in the presence of only 10 µM IPTG, allowing this small number of clones to survive under LCCR4 stress, while 95% of the recovered clones carried the wt sequence EDxxR. Nevertheless, such a low level of RNase induction could not be used in the gradient plates, as the control cells expressing the wt enzyme (that are used for normalization) were growing over the whole distance of the plate.

### HsaCCA E164G displays full CCA-adding activity but altered NTP binding

**Figure 5.**
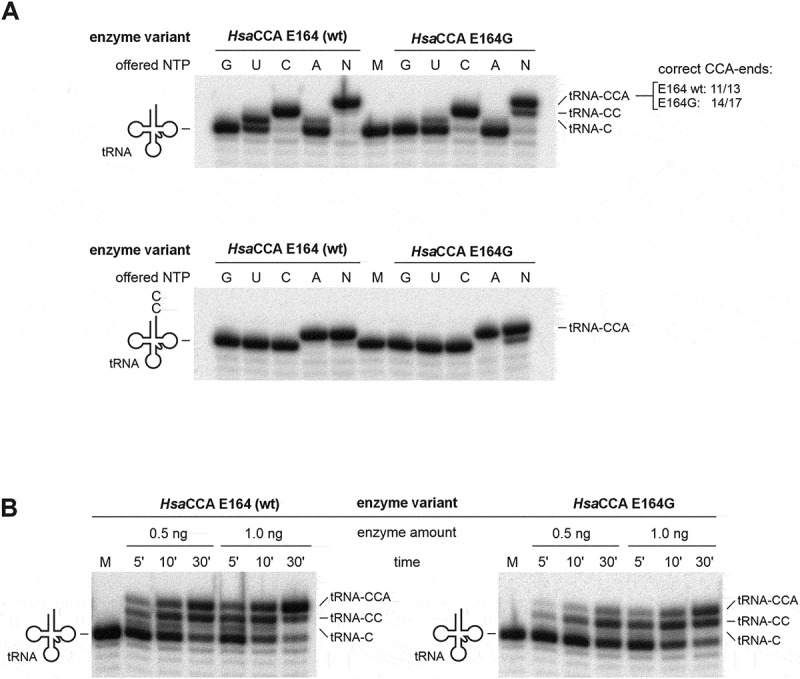
Catalytic properties of *Hsa*CCA wt and variant GDxxR (E164G). (A) *in vitro* nucleotide incorporation analysis with recombinant enzymes. Upper panel: complete CCA-addition on a tRNA substrate lacking the CCA-terminus. When all four NTPs (N) or only CTP (C) are offered, both enzymes show efficient incorporation activity. A partial incorporation activity in the presence of UTP (U) can be detected for both enzymes. Full length reaction products were isolated and analyzed by sequencing; numbers indicate the amount of correct CCA ends in the analysis of 13 and 17 clones, respectively (see also Table 1). Lower panel: A-addition on tRNA-CC. Both enzyme versions readily add the terminal A if NTPs (N) or only ATP (A) is offered. M, mock incubation in the absence of enzyme. (B) Timeresolved product formation in the presence of 0.5 and 1.0 ng enzyme per 20 µl reaction shows a mild decrease in catalytic activity for variant E164G compared to the wildtype situation. M, mock incubation without enzyme.

To characterize the enzymatic features of the unexpected *Hsa*CCA E164G enzyme variant in more detail, the purified recombinant enzyme was tested for *in vitro* CCA-addition and nucleotide specificity. The enzyme was incubated with radioactively labelled yeast tRNA^Phe^ (for CCA-addition) or tRNA^Phe^-CC (for A-addition) in the presence of all four as well as individual NTPs (Figure 5A). For both wild type (wt, E164) as well as *Hsa*CCA E164G, a slight misincorporation of UTP and ATP at position 74 (corresponding to the first C position of the CCA-end) was observed, representing a frequently described *in vitro* artefact for CCA-adding enzymes [8,42,50,51]. The final reaction products (tRNA-CCA) were isolated from the gel, reverse transcribed and cloned. Sequence analysis revealed that for *Hsa*CCA E164G, 14 out of 17 clones carried a correct CCA-end (82%), while 3 clones showed single misincorporations of A instead of C (2×) and U instead of A (1×). For the reaction products of the wt enzyme, 11 out of 13 clones showed correct CCA-addition (85%), and two carried a U misincorporation instead of A (Figure S6). Hence, no increase in misincorporation was detected for *Hsa*CCA E164G, indicating that the templating function was not affected by this mutation (Figure 5A). To investigate the catalytic efficiency of *Hsa*CCA E164G, increasing amounts of mutant as well as wt enzyme were incubated with radioactively labelled tRNA^Phe^ and NTPs for increasing time [15,31,52]. The combined time and enzyme concentration series clearly show that the E164G variant is less active than the wt and only produces complete CCA-ends at higher enzyme concentrations and longer incubation times (Figure 5B). A densitometric analysis of the product bands revealed that both CC- and A-addition were affected (49% and 41% compared to wt, respectively) (Table 1). These results are corroborated by the growth distance of the corresponding transformants in the RNase T selection system (Figure 4D). Hence, compared to the wt enzyme, *Hsa*CCA E164G exhibits a reduction in growth down to 48%.

**Table 1.** Efficiencies of CCA-addition catalysed by *Hsa*CCA wt and *Hsa*CCA E164G. While nucleotide specificity (referring to % correct CCA ends in a full length tRNA) remains unaffected (85% vs. 82%), *Hsa*CCA E164G is less efficient in CC- and A-addition both in vitro as well as in vivo. Product formation rates were determined by densitometric quantification of product bands formed under non-saturating conditions and non-linear regression as mean ± SD (using ImageQuant TL and GraphPad prism 7). *n* = 3.

	nucleotide specificity (%)	CC-addition (pmol min^−1^ ng enzyme)	A-addition (pmol min^−1^ng enzyme)	A repair activity *in vivo* (%)*
*Hsa*CCA wt	85	0.261 ± 0.012	0.128 ± 0.009	100
*Hsa*CCA E164G	82	0.129 ± 0.004	0.052 ± 0.004	52
ratio E164G/wt		49%	41%	

*Estimated from growth distance on IPTG gradient plates (Figure 4).

**Figure 6.**
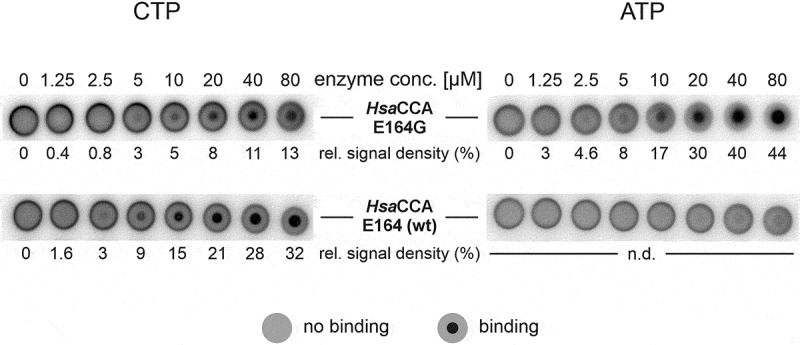
NTP binding of *Hsa*CCA wt and *Hsa*CCA E164G. The DRaCALA assay shows unambiguously that both enzymes interact with CTP (left panel), while only the E164G variant is also able to bind ATP (right panel). Free radioactively labelled NTPs diffuse on the nitrocellulose membrane, leading to a grey circle. Enzyme-bound NTPs remain at the position where the protein is immobilized, resulting in a dark small circle in the centre of the spot. Binding was quantified by relative signal densities of the bound versus the unbound NTP. While the wt enzyme only binds CTP, *Hsa*CCA E164G interacts with CTP as well as ATP, and the existence of both binding-competent states reduces the efficiency of CTP recognition, resulting in reduced relative signal intensity compared to the wt enzyme. As both proteins showed increasing precipitation at higher concentrations, dissociation constants could not be determined.

To investigate how G164 in the amino acid template causes the observed effect on CCA-addition efficiency, the interaction of wt and mutant enzyme with CTP and ATP was analysed. To this end, DRaCALA assays were performed, using radioactively labelled CTP or ATP and an increasing amount of enzyme (Figure 6). Both *Hsa*CCA wt and *Hsa*CCA E164G showed robust interaction with CTP, indicated by the increasing density of the inner spots (correlating with enzyme concentration), where the protein was immobilized on the nitrocellulose membrane. However, CTP binding of *Hsa*CCA E164G seems to be less efficient compared to the wt enzyme, as indicated by the relative signal intensities in the DRaCALA. In contrast, ATP interaction was only detectable for *Hsa*CCA E164G, while the wt enzyme showed no binding. As higher enzyme concentrations could not be used due to protein precipitation, no binding constants could be determined. Yet, the data clearly indicate that the glycine residue in the nucleotide-binding pocket renders the *Hsa*CCA E164G apoenzyme competent to interact with both CTP and ATP, while the wt enzyme is restricted to CTP interaction.

### *In vivo* activity of two different enzyme variants with reduced A addition

**Figure 7.**
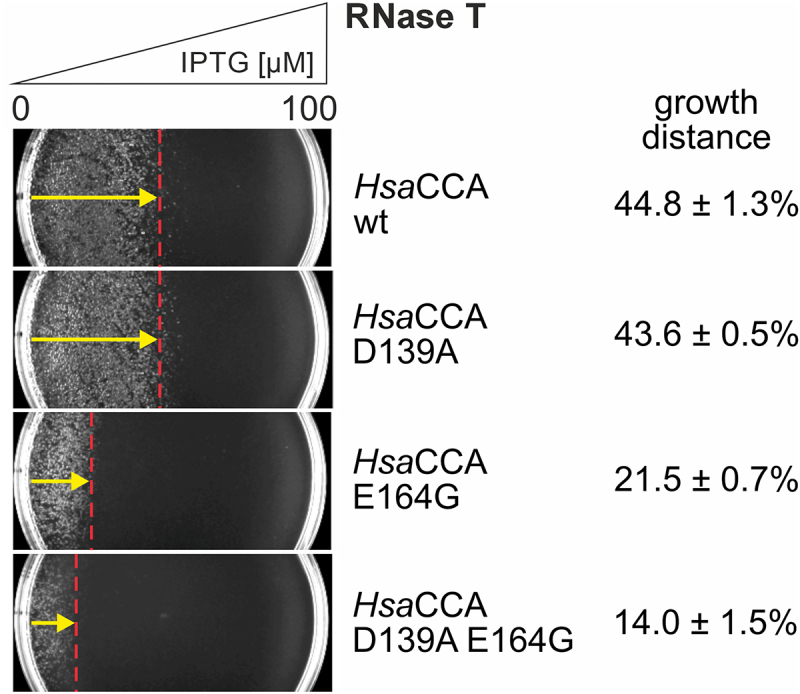
Growth behaviour of *E. coli* expressing *Hsa*CCA variants on RNase T gradient plates. Depicted gradient plates represent one assay of a triplicate analysis (*n* = 3). Compared to *Hsa*CCA wt, *Hsa*CCA D139A does not result in a reduced cell viability in the presence of RNase T and grows up to a distance of 43.6 ± 0.5%. As the *in vitro* A-adding activity of *Hsa*CCA D139A is 15-fold reduced, this unchanged growth behaviour is an indication for an efficient endogenous A-addition backup system by poly(A) polymerase and PNPase. *Hsa*CCA E164G, however, leads to a reduced growth of 21.5 ± 0.7%, indicating that this mutation is more detrimental, and the backup system is not sufficient to restore wt growth. The double variant *Hsa*CCA D139A E164G shows a further growth reduction of down to 14.0 ± 1.5%, suggesting that the combination of both amino acid replacements leads to a further saturation of the A-addition backup.

For class II CCA-adding enzymes, several mutations were reported to specifically affect the incorporation of the terminal A residue [9,10,33,53,54]. Besides the described E164G substitution, point mutations and deletions in the flexible loop element as well as the replacement of aspartic acid by alanine at position 139, located in motif C, interfere with A-addition (Figure S1) [8–10,33]. While the loop mutations completely abolish A incorporation, D139A still catalyzes A-addition, although at a 15-fold reduction compared to the wild type enzyme [33]. Motif C is described as a flexible spring element involved in interdomain movement for switching the enzymes’ specificity from C- towards A-addition [33]. To characterize the efficiency of this reaction step, *Hsa*CCA D139A was introduced into the RNase T system, and cell growth was monitored on IPTG gradient plates. In addition, a combination of the variations D139A and E164G, both exhibiting a reduced A-incorporation, was investigated (Figure 7). Interestingly, *Hsa*CCA D139A leads to a growth phenotype indistinguishable to the wt, with a growth distance of 43.6 ± 0.5% (wt: 44.8 ± 1.3%) across the RNase T gradient, in contrast to *Hsa*CCA E164G (21.5 ± 0.7%; *p* = 0.0001; Figure 7 shows representative gradient plates). The combination of both D139A and E164G, however, shows a synergistic effect, resulting in a growth distance of only 14.0 ± 1.5% that is significantly lower than that of E164G alone (*p* = 0.0015). Hence, although the isolated reduced A-addition of D139A is obviously compensated by auxiliary AMP incorporating activities in the cell, the RNase T system reveals that the activity of these auxiliary enzymes is saturated by the additive effect of E164G in combination with D139A.

### Monitoring the activity of pathogenic variants of the human CCA-adding enzyme

A constantly increasing number of cases representing mutations in the open reading frame of TRNT1, the gene encoding the human CCA-adding enzyme, is reported in the literature [55]. However, in nearly all cases, the molecular effect of these amino acid replacements is not characterized. To demonstrate that our double screening approach allows for a rapid testing of such defects, we investigated the effect of the R99W replacement. This enzyme variant represents one of the most frequent pathogenic mutations in *Hsa*CCA and is linked to SIFD (sideroblastic anaemia, B-lymphocyte immunodeficiency, periodic fevers and delayed development) [55]. Furthermore, R99W occurs together with D163V as a double mutation in two different affected families [56]. Another pathogenic mutation is M158V in motif B, also linked to SIFD. Here, *in vitro* results suggest that it has a negative effect on C-addition, while A-addition is not hampered [53]. Note that the numbering of pathogenic replacements includes the N-terminal mitochondrial import sequence (29 amino acids) [25,55], while in biochemical investigations, this sequence is omitted [10,32–34]. For a consistent numbering, we therefore follow the biochemical scheme, so that R99W corresponds to R70W, D163V is represented by D134V, and M158V corresponds to M129V (Figure S1).

**Figure 8.**
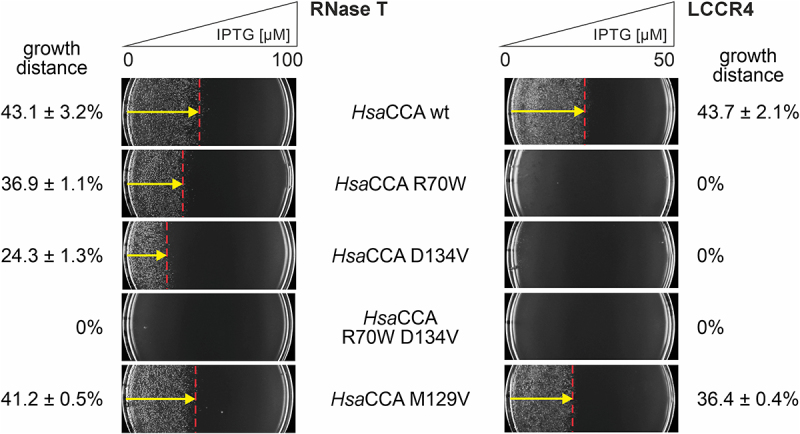
Growth behaviour of *E. coli* host cells expressing *Hsa*CCA carrying disease-causing mutations. Representative plates of a triplicate analysis are shown (*n* = 3). Left panel: monitoring of A-addition in the RNase T system. wt *Hsa*CCA expression leads to a growth distance of 43.1% of the total plate distance, while pathogenic variants R70W and D134V reduce the growth distance down to 36.9% and 24.3%, respectively. A combination of both completely abolishes cell growth. The equally pathogenic mutation M129V shows almost no effect, as the cells grow up to a distance of 41.2%, nearly identical to that of cells expressing the wt enzyme. Right panel: monitoring full CCA-addition in the LCCR4 system. Due to the increased stringency, R70W, D134V and R70W/D134V enzyme variants cannot restore viability in *E. coli*, indicating that C-addition is strongly affected. Expression of *Hsa*CCA M129V, however, leads to considerable growth to up to 36.4%. Here, a slight reduction compared to the wt enzyme is visible. Hence, in this variant, A-addition is similar to the wt level, while C-addition is affected, but not as severe as in the other tested pathogenic variants.

To identify the individual effects of these replacements, the amino acid exchanges were tested separately in both RNase T- (A-addition) and RNase LCCR4 (CCA-addition) systems (Figure 8). As in the previous tests, RNase T was induced with up to 100 µM IPTG, while for LCCR4, only 50 µM IPTG was used, as this system exhibits an increased stringency due to the required complete CCA-end restoration. Both *Hsa*CCA R70W and D134V show slight but clearly visible growth retardation in the presence of RNase T, resulting in 36.9% and 24.3% of growth distance compared to wt with 43.1% (Figure 8, left panel). The combination of both, however, was not viable, even under very low RNase T stress. In contrast, *Hsa*CCA M129V showed a growth behaviour similar to that of the wt enzyme (41.2% vs. 43.1%).

In the LCCR4 system, neither R70W nor D134V or the combination of both replacements show any growth, indicating that these mutations strongly affect C-incorporation. *Hsa*CCA M129V, however, exhibits a somewhat reduced growth relative to the wt enzyme (36.4% vs. 43.7%), and the combined evaluation of both systems indicates that this mutation is much less affected than the other two tested, with an A-addition similar to the wt enzyme (visible in RNase T system) and a slight but detrimental decrease in C-incorporation (visible in LCCR4 system).

## Discussion

tRNA nucleotidyltransferases are fascinating RNA polymerases with a unique mode of nucleotide selection based on an internal amino acid template, where amino acid side chains form Watson-Crick-like hydrogen bonds to the incoming nucleotides [6,57]. While these enzymes are extensively studied *in vitro*, an *in vivo* analysis bears the advantage that such a system is closer to natural conditions, allowing a more stringent investigation of the enzyme of interest. A previously established selection system is based on RNase T-catalysed removal of the terminal A residue of the CCA-end and therefore monitors only A-addition/restoration in an *E. coli cca* knockout strain [30,31]. Here, we describe a new approach that represents a more demanding screening/monitoring system. It is based on the expression of LCCR4, a 3’exonuclease identified in *T. brucei* [23]. As this enzyme removes whole CCA-ends from tRNAs, the screening system monitors for complete CCA-end restoration, resulting in an increased selection pressure on the enzymes to be tested. Accordingly, in the screening of an *Hsa*CCA pool carrying a randomized amino acid template, the LCCR4 system retrieved predominantly the wt sequence EDxxR (at positions 164, 165 and 168, respectively) and only GDxxR (5%) as an alternative, while DDxxR was not identified, in contrast to the RNase T-based system (Figure 4, S4). This finding is in agreement with the fact that A-addition is less demanding for the tRNA nucleotidyltransferase than a complete CCA-addition, where a structural rearrangement of the nucleotide-binding pocket and the amino acid template region is required for switching the specificity from CTP towards ATP [6,9,29,33].

Previously, it has been shown that in tRNA nucleotidyltransferases from human, *Thermotoga maritima* and *Aquifex aeolicus*, alanine replacements at this position (E164A, D173A, E148A, respectively) lead to a dramatic reduction in enzymatic activity [10,29,58]. This observation is supported by our *in vivo* data on variant *Hsa*CCA E164A which indicate that the alanine replacement cannot promote growth when challenged with RNase T-mediated tRNA end hydrolysis in *E. coli* (Figure 4D). Although a substitution of alanine with glycine represents a minor change, both *in vivo* as well as *in vitro* results manifest E164G as an enzyme variant with full and precise CCA-adding activity. Yet, compared to the wt enzymes, its efficiency is reduced, and the product formation rates imply an impact on both CC- as well as A-addition (Figure 5, Table 1). The finding that A-addition is slightly more affected than CC-addition (41% residual activity vs. 49%) supports the idea that the nucleotide-binding pocket of the apo enzyme is predefined for CTP accommodation, as its conformation is similar to that of the enzyme in CTP-bound state [6]. This is further corroborated by the binding behaviour of *Hsa*CCA wt, where a selective interaction with CTP could be observed, while ATP is not bound (Figure 6). In contrast, the enzyme variant with a glycine residue at position 164 interacts with both CTP as well as ATP.

Glycine is described as a crucial mediator of flexibility in enzyme active sites [59,60]. In particular, this amino acid can account for common patterns in proteins that accommodate nucleotides within a binding pocket [61,62]. In CCA-adding enzymes, glycine residues have already been identified as key features important for A-addition, like G70 in the flexible loop of *Eco*CCA [54]. As glycine carries the smallest side chain of all amino acids, it enlarges the space in the nucleotide-binding pocket of *Hsa*CCA E164G. Hence, while it does not interact with the templating arginine residue 168 for proper positioning (as does the original glutamate, see Figure 4A) [6,47,57], it probably allows for an increased structural freedom of R168, resulting in a binding pocket that readily adopts CTP- as well as ATP-binding conformations. Due to the competition of ATP- and CTP-binding states in *Hsa*CCA E164G, the efficiency of CTP interaction is reduced compared to the wt enzyme, resulting in a lower relative signal intensity in the DRaCALA test (Figure 6). This competition in nucleotide selection contributes to the reduced A- and CC-adding activity of *Hsa*CCA E164G (Table 1).

Yet, the E164G variant exhibits a fidelity in CCA-addition almost identical to that of the wt enzyme (82% vs. 85%; Table 1, Figure 5A), and C and A residues are specifically incorporated at the correct CCA positions. Hence, additional safeguard systems avoid misincorporation of a bound ATP. In this context, a β-turn in the catalytic core plays an important role, as it recognizes the growing CC-end of the tRNA and positions it for proper A-addition [29,39]. Furthermore, the bound tRNA primer triggers a rearrangement of the catalytic core so that only the correct CMP is transferred to its 3’-end. Such domain movements were already described for several CCA-adding enzymes, where they are involved in proper nucleotide selection [9,10,33,58,63]. A mutation that interferes with this domain movement is D139A in the hinge element motif C [33]. As a consequence, the CCA-adding enzyme is reduced in its A-adding activity. Yet, in the RNase T system, *Hsa*CCA D139A leads to a growth phenotype indistinguishable from cells expressing the wt *Hsa*CCA (Figure 7). An explanation for this surprising finding is a functional overlap of the CCA-adding enzyme, the bacterial poly(A) polymerase (PAP) and polynucleotide phosphorylase (PNPase), leading to the restoration of the missing A residue at the tRNA 3’-end [28]. PAP adds a series of A residues to the truncated tRNA ending with CC, and the exonucleolytic activity of PNPase trims this poly(A) tail down to the functional CCA-end. Alternatively, it is conceivable that the negative *in vitro* effect of the D139A replacement [33] is compensated *in vivo* by proteins supporting the activity of the CCA-adding enzyme. Here, especially chaperone systems like GroEL or DnaK could assist the correct folding of the mutated enzyme, resulting in an enzymatic activity comparable to wt [64–66]. Furthermore, it is known that the Sm-like protein Hfq stimulates the activity of the CCA-adding enzyme by presenting tRNA substrates to the enzyme [67].

However, if a second mutation E164G is introduced, which also affects A-addition, the compensatory capabilities of the cell are not sufficient to restore a wt-like activity of the enzyme, and the cells are highly sensitive to RNase T-catalysed tRNA damage. As a result, cell growth is only observed at IPTG gradient positions with very low RNase T induction (Figure 7). Hence, this *in vivo* system reveals that the effect of an altered amino acid template GDxxR is more dramatic than the decreased functionality of the spring element in motif C, where a somewhat reduced interdomain movement is tolerated to a certain extent.

A further interaction postulated for E164 is a salt bridge between the glutamate and a conserved arginine residue at position 105, located in the flexible loop element [10,47]. It has been assumed that this interaction triggers the rearrangement of the templating amino acids to recognize ATP in place of CTP. However, the formation of this salt bridge is not possible with G164. Yet, the enzyme variant can switch its specificity in a coordinated fashion for proper CCA synthesis. Surprisingly, our selection systems clearly show that a correct and efficient CCA-addition requires an arginine residue at position 105, independent of the nature of the first template position (Figures 2B, 3, S3). Thus, it is evident that the role of R105 exceeds a direct function in rearranging the amino acid template. The structural analysis of the corresponding enzyme from *Thermotoga maritima* (*Tma*CCA) suggests that the flexible loop region serves as a connector element between motif D and the above mentioned β-turn element, where several loop residues (including a critical tyrosine at a position corresponding to 105 in *Hsa*CCA) play a crucial role in AMP but not CMP incorporation [29]. The β-turn itself is involved in the proper positioning of the tRNA 3’end for nucleotide incorporation, and the interplay of the flexible loop with both the amino acid template and this turn element seems to coordinate the individual steps of polymerization and nucleotide specificity switch in CCA synthesis.

Nevertheless, the reduced efficiency of *Hsa*CCA E164G compared to the wt enzyme indicates that a GDxxR template has a detrimental effect on CCA-addition. As the NTP-selecting R residue has an increased rotational freedom in GDxxR, it is conceivable that this flexibility reduces the speed of polymerization, as the enzyme has to wait until the correct nucleotides are bound in a sequential fashion, while misincorporation is avoided due to the safeguard mechanisms described above. Consequently, no wt CCA-adding enzyme with a GDxxR template is described so far. Yet, the selected *Hsa*CCA E164G variant provides important insights into the mechanism of CCA-addition, especially for the communication of the flexible loop region with the templating amino acids and further parts of the enzyme. Furthermore, the challenging *in vivo* system revealed that some mutations (D139A) affecting CCA-addition *in vitro* are compensated by safeguard mechanisms of the cell (redundant activities of PAP and PNPase, chaperone systems assisting protein folding or tRNA-presenting Hfq), while others (E164G) are more dramatic and, consequently, not amended.

The only tolerated variation at the first template position is aspartic acid, the second acidic amino acid, resulting in DDxxR. Interestingly, the situation is different in CC- and A-adding enzymes – tRNA nucleotidyltransferases with complementing partial activities [9,68,69]. Whereas in A- as well as in CCA-adding enzymes exclusively EDxxR and DDxxR templates are found, CC-adding enzymes show a high variation at the first template position (Figure S7). As this type of tRNA nucleotidyltransferase stops polymerization after the addition of two C residues, a specificity switch to adjust the nucleotide-binding pocket for AMP incorporation is not required. Hence, both flexible loop (missing in CC-adding enzymes [9]) and first template position (high sequence variability, Figure S7) are dispensable in CC-adding enzymes.

The fact that we did not retrieve other amino acids than the wildtype at positions D165 and R168 underscores the importance of precise hydrogen bonding between these templating amino acids and the incoming nucleotide [6,57]. Accordingly, an alanine substitution at position R134 in *Eco*CCA (corresponding to R168 in *Hsa*CCA) was unable to promote growth in the RNase T system [30]. These findings are corroborated by the fact that no deviations are observed at these positions in tRNA nucleotidyltransferases with full or partial CCA-adding activity.

While a constantly increasing number of pathogenic mutations in the gene for the CCA-adding enzyme is observed, only a few of these disease-related enzymes are characterized concerning their activity. From mutational analyses not related to human diseases, it is known that most mutations in CCA-adding enzymes lead to a defect in A-addition, while C-incorporation is much less affected [8,10,29,34,54,58]. Our double screening approach allows for a rapid investigation of pathogenic enzyme variants to identify their molecular effects on A- or C-incorporation.

The results of the tested pathogenic mutations R70W, D134V and M129V in both RNase T and LCCR4 systems corroborate the described *in vitro* activities of these enzyme variants. R70W, one of the most frequent pathogenic mutations in *Hsa*CCA [55], is located in the β-turn involved in primer positioning during elongation of the tRNA [29,39]. The importance of a correct positioning of the tRNA 3’end is demonstrated by a reduced growth phenotype in the RNase T system, indicating that A-addition is affected. However, the effect of R70W on C-addition is even more dramatic, as the cells expressing *Hsa*CCA R70W in the LCCR4 system show no growth at all. A similar result is obtained for *Hsa*CCA D134V, located in motif C, where a spring element is involved in switching the enzyme specificity from CTP towards ATP [33]. Again, this mutation affects not only A-addition, but also C-addition, and it is likely that this motif also contributes to structural rearrangements during the serial addition of the two C-residues. If both R70W and D134V are combined, the detrimental effect is so dramatic that even A-addition is severely impaired so that no growth is observed in the RNase T system.

The last pathogenic mutation M129V, located in motif B, was described to exhibit a reduced C-adding activity and a rather normal A incorporation [53]. Our combined RNase T and LCCR4 systems support these findings and show a reduced growth phenotype only in the LCCR4 system, indicating that A-addition is almost at wt level, while C-addition is reduced and obviously the molecular cause of the pathogenic effect.

In conclusion, we present a method that allows for the *in vivo* monitoring of the addition of complete CCA-ends on the tRNA pool of an *E. coli* reporter strain. Especially, when combined with the screening system for terminal A-addition, this approach has a strong advantage over a mere rational design. The selection and characterization of the GDxxR template variation in a tRNA nucleotidyltransferase is such an example, as it is highly unlikely that such a variant would have been designed intuitively in a rational approach. This *in vivo* monitoring system is also very useful for a rapid screening of the increasing number of disease-related mutations in the corresponding human gene TRNT1 [25,30], and the obtained results demonstrate the reliability of both systems in the analysis of pathogenic enzyme variants. In addition, the activity of newly identified CCA-adding enzymes or variants resulting from ancestral sequence reconstructions can be investigated and compared to modern enzyme counterparts [31].

## Supplementary Material

-)Supple fig.docx

## Data Availability

Lists of utilized oligonucleotides as well as selected coding sequences from the randomized pools of CCA-adding enzyme ORFs are available in the Supplementary Data online.
