# Supplementary material for: Two complementing in vivo selection systems based on CCA-trimming exonucleases as a tool to monitor, select and evaluate enzymatic features of tRNA nucleotidyltransferases
Source: RNA Biol. 2025 Jan 20;22(1):1–14. doi: 10.1080/15476286.2025.2453963 (PMC11784652; doi:10.1080/15476286.2025.2453963)
Supplement: -)Supple fig.docx [file KRNB_A_2453963_SM7040.docx]

**Two complementing *in vivo* selection systems reveal an alternative amino acid composition in the nucleotide-binding site of a tRNA nucleotidyltransferase**

Karolin Wellner, Josefine Gnauck, Dorian Bernier, Stephan H. Bernhart, Heike Betat and Mario Mörl

# Supplementary Information


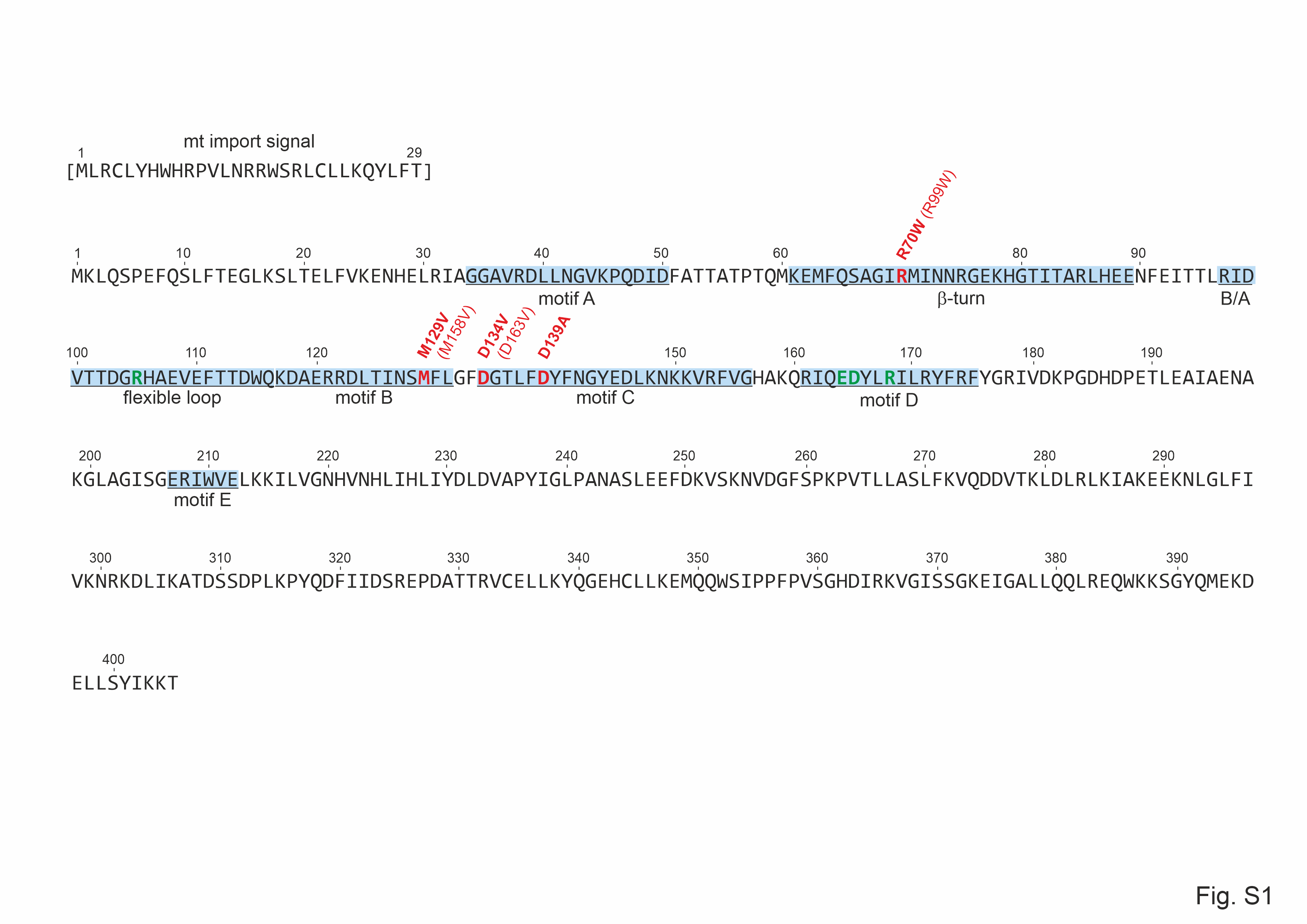


**Figure S1. Sequence of the human CCA-adding enzyme and investigated amino acid replacements.** Position numbering corresponds to the one used in biochemical experiments and does not include the mitochondrial target sequence (shown in square brackets). The numbering in clinical studies includes this region, resulting in a difference of 29 amino acids. Conserved catalytic core elements are indicated in light blue, positions subjected to randomization and selection are shown in green. Tested individual point mutations are indicated in red (for pathogenic positions, the numbering used in clinical studies is given in brackets).


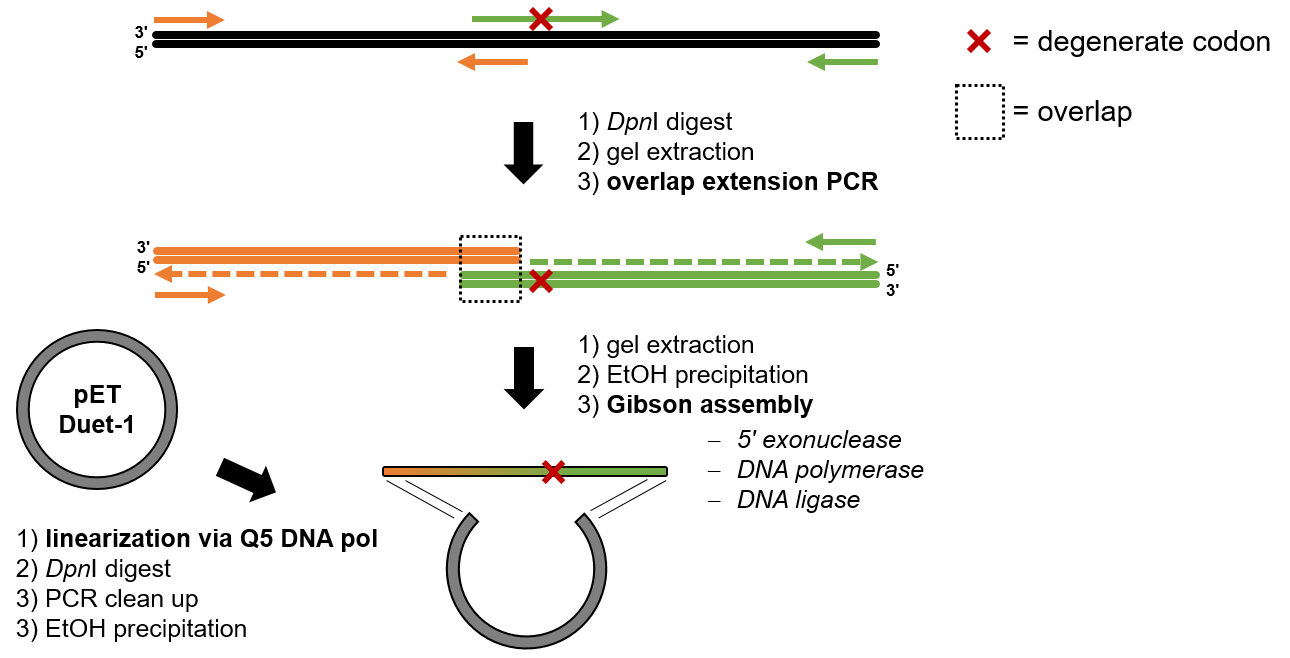


**Figure S2. Library preparation in pET Duet-1 as a vector system.** Degenerate codons were introduced by PCR-based extension of a corresponding oligonucleotide (green with red cross), amplifying the 3’-part of the coding sequence. In a second PCR, the 5’-part of the coding sequence was amplified (orange). In an overlap extension, both PCR products were fused. The resulting product was inserted into pET Duet-1 by Gibson assembly using the NEBuilder HiFi DNA assembly (New England Biolabs) after linearization of the vector DNA with Q5 DNA polymerase (New England Biolabs).


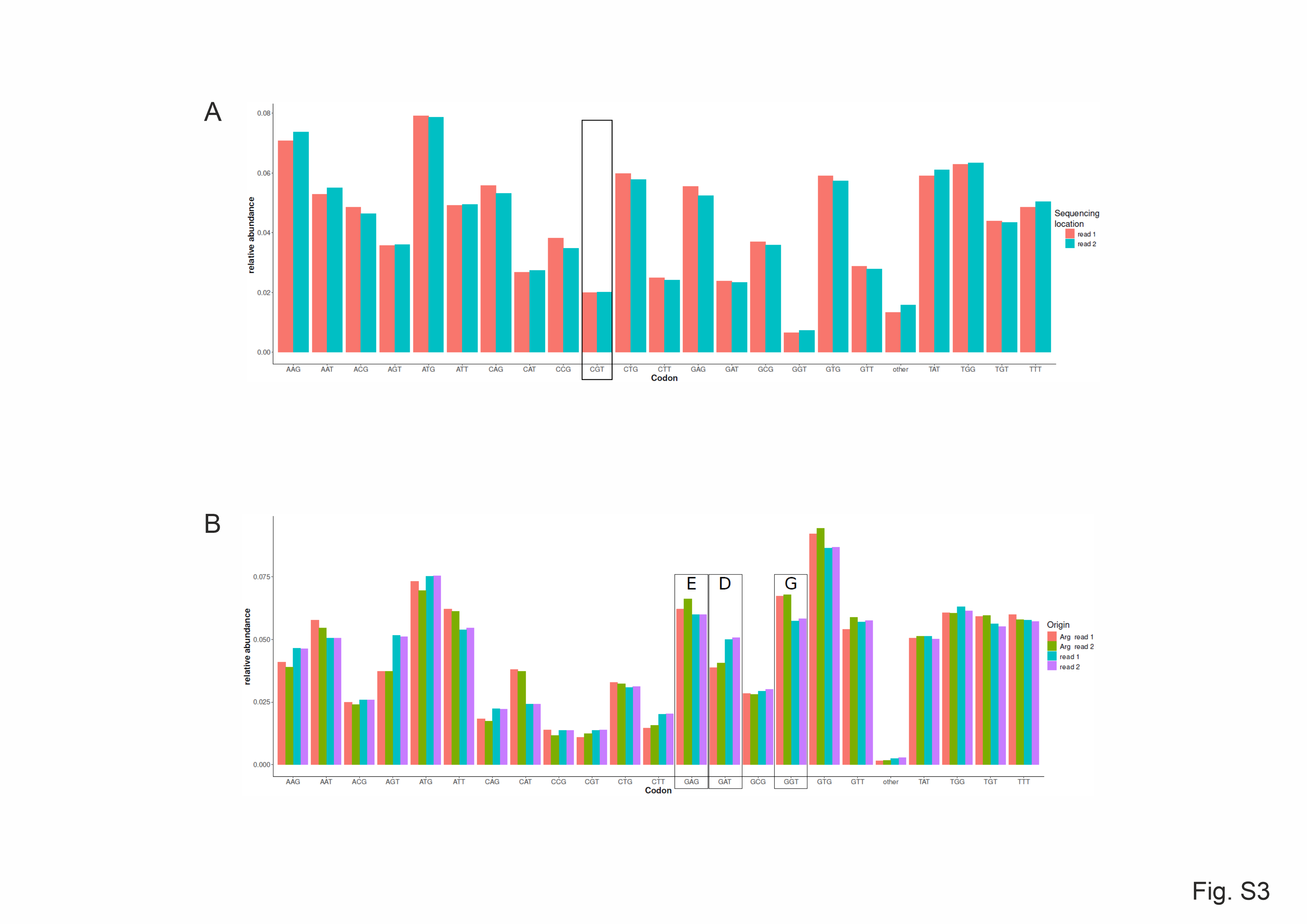


**Figure S3. Codon abundance in randomized regions determined by Amplicon sequencing.** **(A)** Relative codon abundance in the library of randomized codon position 105. Read 1 (n = 68,496) of the paired end sequencing is indicated in red, read 2 (n = 59,920) in cyan. The boxed arginine codon (CGT) is among the least abundant inserted codons. **(B)** Relative abundance in the library of randomized codons at position 164. Read 1 (n = 68,496) of the paired end sequencing is indicated blue, read 2 (n = 59,920) in violet. Reads containing in addition the R codon at position 105 are also shown: read 1 (n = 1,367) is labeled in red, read 2 (n = 1,207) in green.


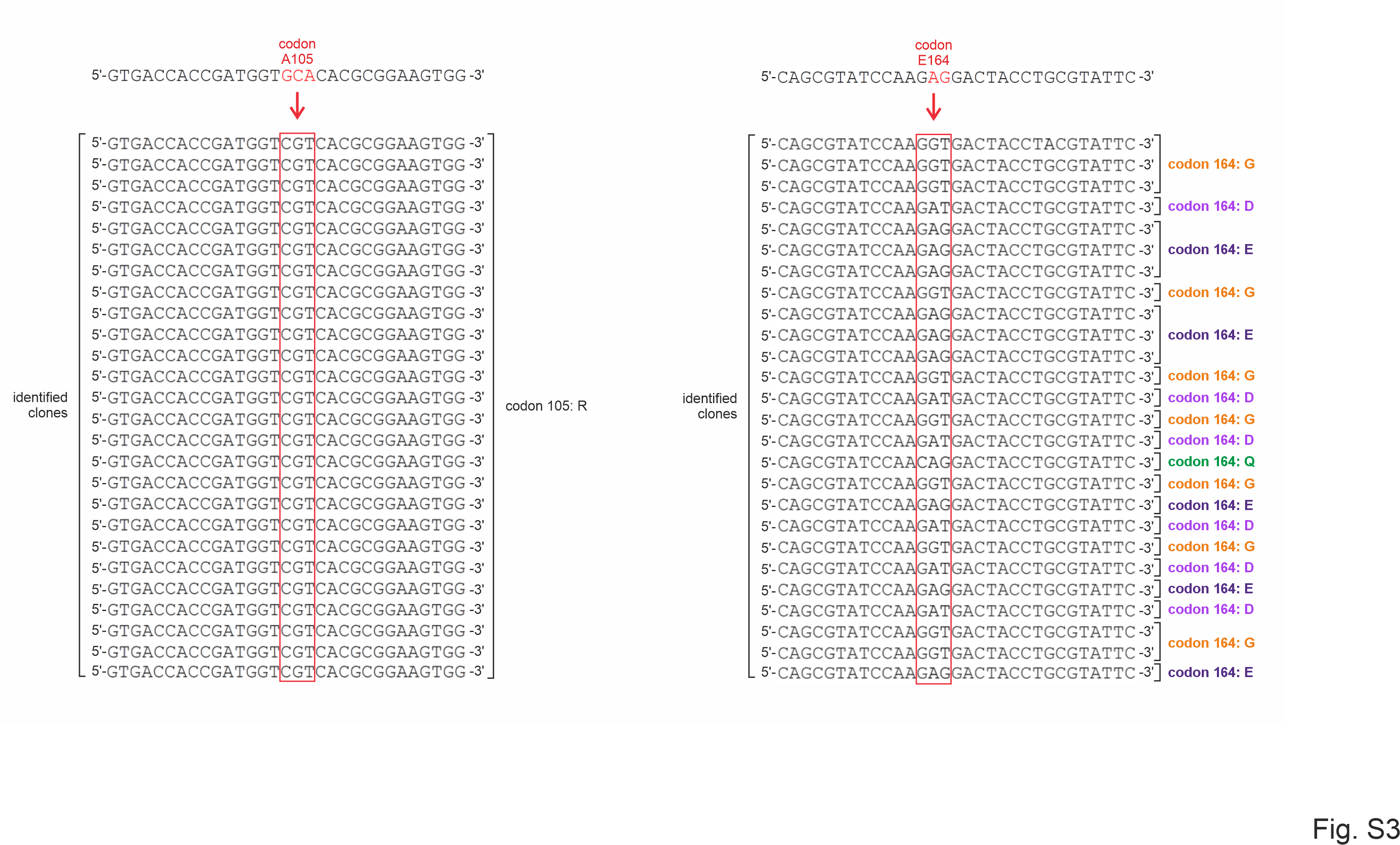


**Figure S4. *In vivo* selection of an *Hsa*CCA-encoding sequence library with simultaneous randomizations at positions 105 and 164.** Selection was performed in the RNase T system, screening for A-restoration. The starting sequence contained an alanine codon 105 and a glutamate codon 164. At position 105, the retrieved sequences contained exclusively arginine codons (left panel). At position 164, the acidic residues glutamate (deep purple; corresponding to the wt situation) and aspartate (purple) were identified in 58% of the sequences. Interestingly, glycine appeared in 38% (orange), and in 4%, glutamine (green) was found.


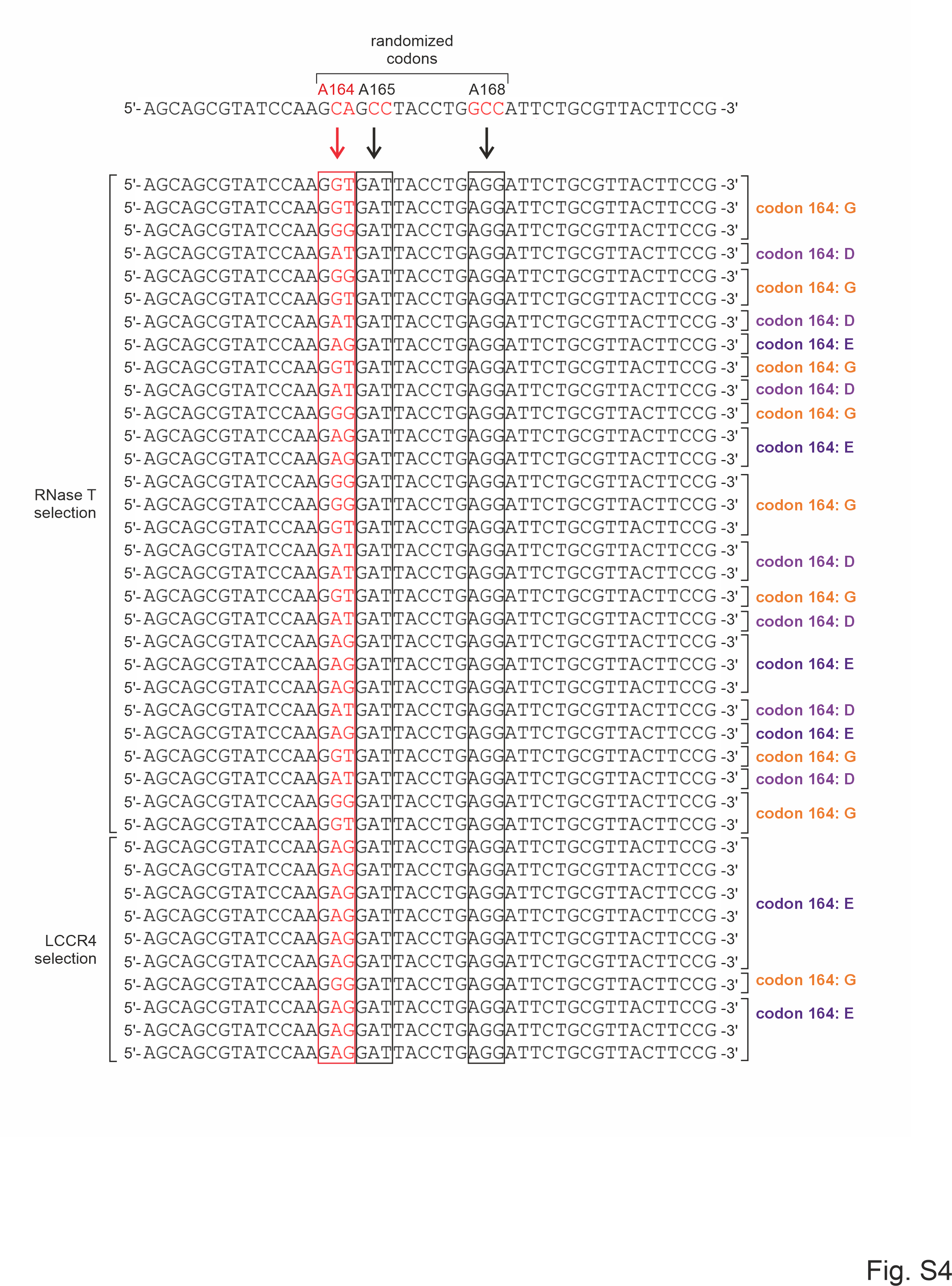


**Figure S5. Randomization of the complete amino acid template EDxxR in *Hsa*CCA and selection in the RNase T (A-restoration) and the LCCR4 system (CCA-restoration).** In both selection systems, position 165 (aspartate) and 168 (arginine) were invariable (black frames). Position 164 (red frame), however, showed different frequencies. In A-restoration (RNase T selection system), glutamate (E, deep purple; 32%), aspartate (D, purple; 36%) and glycine (G, orange; 32%) were retrieved after selection (percentage values are normalized for codon frequency in the input library). The LCCR4 selection system is more demanding, as restoration of the complete CCA-end is required. Here, predominantly E164 appeared (95%), while G164 was found only once (5%; normalized values). Hence, while both systems retrieve the glycine residue at position 164, LCCR4 activity leads to a higher selection pressure that favors appearance of the wt amino acid template. Taken together, position 164 is the only position in the amino acid template where certain variations are compatible with full and high fidelity CCA-addition, although at reduced efficiency. For both selection systems, randomization was carried out on an ORF carrying AAxxA codons.


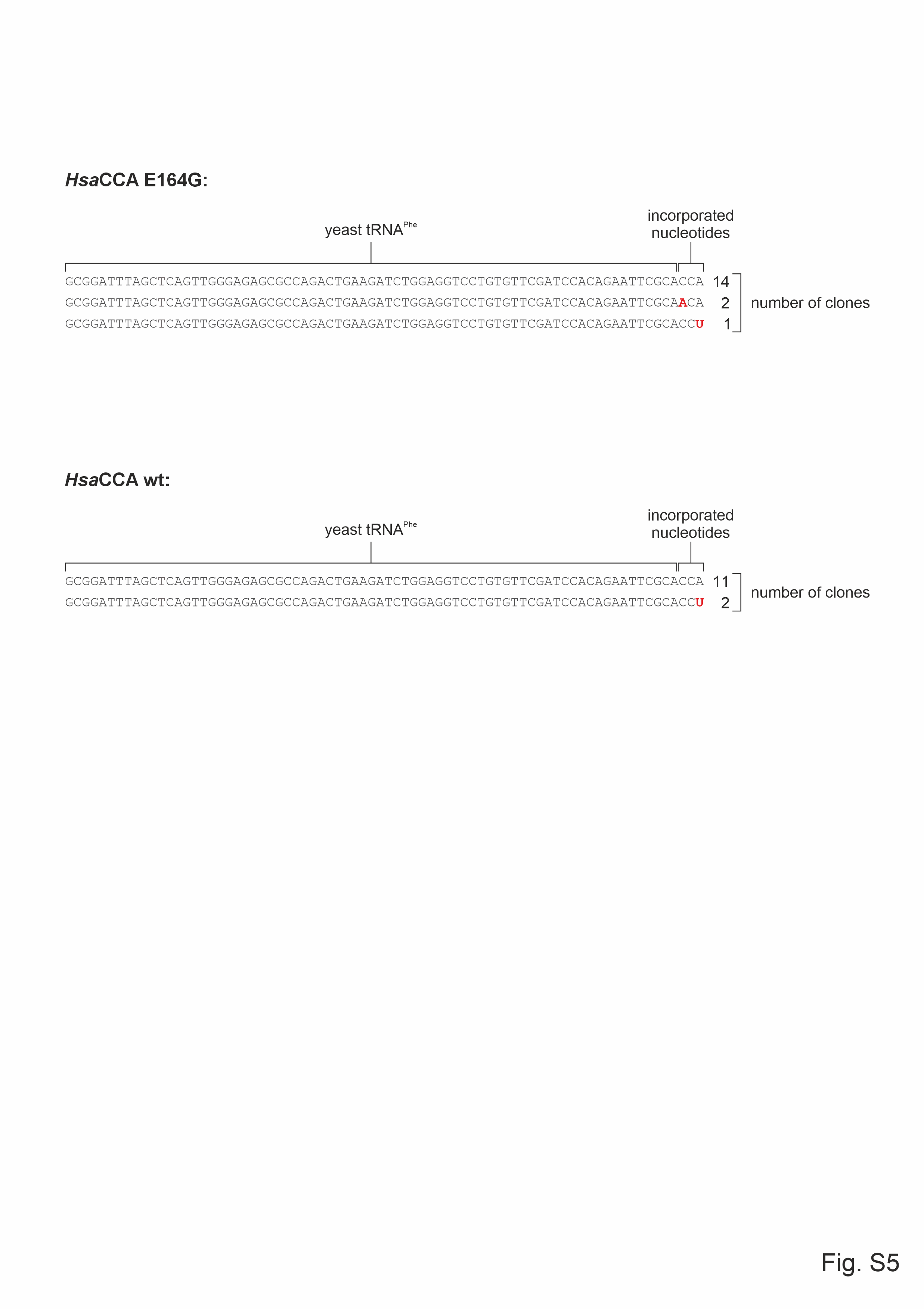


**Figure S6. cDNA sequences of analyzed individual clones of *in vitro* reaction product yeast tRNA^Phe^.** Upper panel: In 14 of 17 analyzed clones, *Hsa*CCA E164G added a correct CCA end. In 2 clones, ACA instead of CCA was added, and one clone showed a misincorporation of CCU. Lower panel: For the wt enzyme, 11 analyzed clones carried a correct CCA end, and two exhibited a misincorporation of CCU. Misincorporated nucleotides are indicated in red.


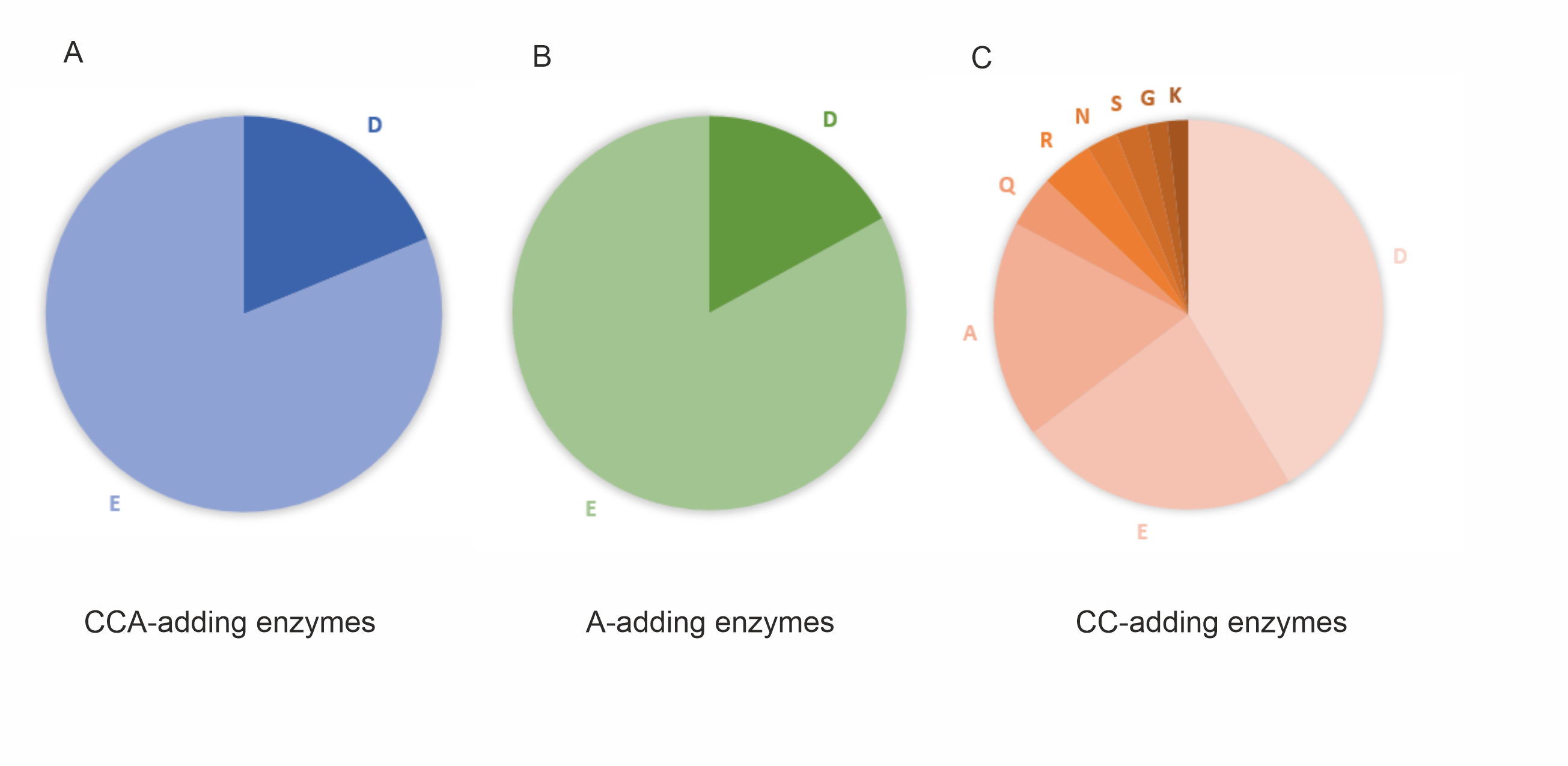


**Figure S7. Distribution of amino acid residues at the first template position in the binding pocket of verified and predicted bacterial tRNA nucleotidyltransferases.** The incoming nucleotides CTP or ATP are recognized by the formation of Watson-Crick-like hydrogen bonds between the edges of the bases and conserved residues of the binding pocket (E164, D165 and R168 in motif D of *Hsa*CCA, see Fig. 3A and B). **(A)**CCA-adding enzymes (293 sequences analyzed) and **(B)**A-adding enzymes (94 sequences analyzed) carry exclusively E and D at the first position of the amino acid template (corresponding to position E164 in *Hsa*CCA). **(C)**In CC-adding enzymes (116 sequences analyzed), this position is highly variable. Besides E and D, also A, Q, R, N, S, G and K are found. Obviously, this position is dispensable for C-addition, while it is essential for terminal A incorporation.

Table S1. List of oligonucleotides used for library preparation.

| **DNA library preparation:** |  |
| --- | --- |
| **primer code** | **sequence 5′-3′** |
| **for_HsaCCA_opt_R105A** | CCGATGGTGCACACGCGGAAGTGGAATTTACC |
| **rev_HsaCCA_opt_R105A** | CTTCCGCGTGTGCACCATCGGTGGTCACGTC |
| **for_HsaCCA_opt_105NNK** | CACCGATGGTNNKCACGCGGAAGTGG |
| **rev_HsaCCA_opt_105NNK** | CTTCCGCGTGMNNACCATCGGTGGTCACG |
| **for_Hsa105_F1** | CCGCGAAATGAATTCCAAACGCAAAG |
| **rev_Hsa105_F1** | CCATCGGTGGTCACGTCAATAC |
| **Hsa105_22cTrickNDT_F2** | GTATTGACGTGACCACCGATGGTNDTCACGCGGAAGTGGAATTTAC |
| **Hsa105_22cTrickVHG_F2** | GTATTGACGTGACCACCGATGGTVHGCACGCGGAAGTGGAATTTAC |
| **Hsa105_22cTrickTGG_F2** | GTATTGACGTGACCACCGATGGTTGGCACGCGGAAGTGGAATTTAC |
| **rev_Hsa105_F2** | TTGGATACGCTGCTTCGCGTGG |
| **forQ5_Hsa105_pETDuet** | CGTTTCGTTGGCCAC |
| **revQ5_Hsa105_pETDuet** | CAGCGATCTTTGCGTTTG |
| **Hsa164_22cTrickNDT_F3** | CGTTTCGTTGGCCACGCGAAGCAGCGTATCCAANDTGACTACCTGCGTATTCTGC |
| **Hsa164_22cTrickVHG_F3** | CGTTTCGTTGGCCACGCGAAGCAGCGTATCCAAVHGGACTACCTGCGTATTCTGC |
| **Hsa164_22cTrickTGG_F3** | CGTTTCGTTGGCCACGCGAAGCAGCGTATCCAATGGGACTACCTGCGTATTCTGC |
| **rev_Hsa164_F3** | CGCAACATCCAGGTCGTAAATCAG |
| **forQ5_Hsa164_pETDuet** | CCTGATCCACCTGATTTACG |
| **for_Q5sub_HsaCCA_optAAxxA** | CCTGGCCATTCTGCGTTACTTCCGTTTTTATG |
| **rev_Q5sub_HsaCCA_optAAxxA** | TAGGCTGCTTGGATACGCTGCTTCGC |
| **for_HsaCCA_opt_105up** | CTTCGAGATCACCACCCTGCG |
| **for_HsaCCA_opt_RVK(AAxxA)** | CACGCGAAGCAGCGTATCCAARVKRVKTACCTGRVKATTCTGCGTTACTTCCGTTTTTATGG |
